# Multidisciplinary management of pregnancy-associated breast cancer: a clinical pharmacy perspective on chemotherapy and perinatal outcomes

**DOI:** 10.3389/fonc.2026.1918932

**Published:** 2026-08-13

**Authors:** Haoran Liu, Jing Jin, Yanmin Deng, Xianli Wang, Tao Zeng

**Affiliations:** Obstetrics & Gynecology Hospital of Fudan University, Shanghai Key Lab of Reproduction and Development, Shanghai Key Lab of Female Reproductive Endocrine Related Diseases, Shanghai, China

**Keywords:** chemotherapy, clinical pharmacist, maternal-fetal outcomes, MDT management model, pregnancy-associated breast cancer

## Abstract

**Background:**

Pregnancy-associated breast cancer (PABC) is increasing globally and often presents at advanced stages with aggressive features, requiring chemotherapy that balances maternal efficacy and fetal safety. Although multidisciplinary team (MDT) management is recommended, real-world pharmacotherapy data, especially involving clinical pharmacists in Asian populations, are limited.

**Objectives:**

This study aimed to describe the clinical characteristics, chemotherapy regimens, premedication strategies, and maternal-fetal outcomes of PABC managed within an MDT framework, offering a clinical pharmacy perspective on medication management and safety.

**Methods:**

This retrospective, single-center, cross-sectional study included patients diagnosed with PABC between January 2019 and December 2025 who received chemotherapy with clinical pharmacist involvement. Demographic characteristics, pathological findings, chemotherapy regimens, premedication protocols, adverse reactions, and maternal-fetal outcomes were extracted from the electronic medical record system by two independent investigators, with discrepancies resolved through consensus.

**Results:**

A total of 28 patients were included. The median age at diagnosis was 32.41 ± 3.80 years. The majority of patients (64.29%) were diagnosed during the second trimester, and Stage II was the most common pathological stage (39.29%). Under the multidisciplinary model, the predominant pregnancy chemotherapy was anthracycline, cyclophosphamide, and docetaxel (EC-T), administered to 35.71% of patients. Regimen selection and premedication were routinely optimized. Adverse events managed through pharmacist-involved monitoring included leukopenia (7.14%) and severe myelosuppression (7.14%). Pharmacists also provided fetal safety counseling, growth guidance, and parental reassurance on long-term offspring outcomes, embedding pharmaceutical expertise into multidisciplinary care. Regarding maternal and fetal outcomes, 79.31% of patients delivered by cesarean section, 53.57% had full-term deliveries, and the mean neonatal birth weight was 2730 ± 497 g. Low birth weight occurred in 28.57% of newborns, and 17.86% required admission to the Neonatal Intensive Care Unit (NICU).

**Conclusion:**

This study provides real-world evidence for the multidisciplinary management of PABC. In this small sample, second- and third-trimester chemotherapy appeared to be associated with acceptable maternal-fetal outcomes. Integration of clinical pharmacists into the MDT model facilitated evidence-based regimen design, individualized premedication strategies, and proactive adverse event monitoring. These preliminary findings support the feasibility of pharmacist-involved MDT care in PABC, though validation in larger prospective studies with long-term offspring follow-up is needed.

## 1 Introduction

Pregnancy-associated breast cancer (PABC) is defined as breast cancer diagnosed during pregnancy or within one year postpartum. With a pooled global incidence of approximately 19.2 cases per 100,000 deliveries, PABC is relatively rare (1); however, its incidence has shown a slow upward trend in recent decades, driven in part by evolving social factors such as delayed childbearing (1, 2). Studies consistently demonstrate that PABC is typically diagnosed at a more advanced stage than non-pregnancy-associated breast cancer, largely because pregnancy-related physiological changes in the breast complicate clinical examination and limit the utility of imaging modalities (2, 3). Furthermore, PABC molecular subtypes exhibit markedly aggressive features, with an increased proportion of hormone receptor-negative tumors; notably, triple-negative breast cancer accounts for 24.4% to 41.3% of cases (4, 5), and upregulation of genes associated with cell proliferation and immune regulation further contributes to this aggressive phenotype (6). These biological characteristics underscore the central role of systemic chemotherapy in the management of most PABC patients.

Chemotherapy during pregnancy necessitates a delicate balance between maternal therapeutic efficacy and fetal safety. Anthracycline-based regimens remain the cornerstone of gestational breast cancer treatment, with taxanes increasingly incorporated into clinical practice (7). Current NCCN guidelines (2026) recommend an anthracycline plus cyclophosphamide backbone for second- and third-trimester chemotherapy, followed by sequential weekly paclitaxel (8). The fetal safety of these agents is well established, with major congenital malformation rates remaining at population baseline following *in utero* exposure (3, 7, 8). However, pregnancy-induced pharmacokinetic changes—including expanded blood volume, altered hepatic enzyme activity, and increased renal clearance—may reduce systemic drug exposure, potentially compromising therapeutic efficacy (5, 9). Targeted and endocrine therapies remain contraindicated in pregnancy owing to documented fetal toxicity or insufficient safety data (10).

Effective treatment and management of PABC is now widely recommended within a multidisciplinary team (MDT) framework. However, real-world data remain scarce regarding pharmacotherapy decision-making, clinical pharmacist-led pharmaceutical interventions, and maternal-fetal outcomes in this population—with studies involving Asian populations being particularly limited. This study systematically describes the clinical pharmacist’s participation in chemotherapy regimen design, dose adjustment, premedication optimization, and fetal safety surveillance within the MDT framework. Against this backdrop, we conducted a retrospective cross-sectional analysis of PABC patients managed by an MDT with full clinical pharmacist involvement. This study aimed to characterize the clinical and pathological features of these patients, evaluate the appropriateness and safety of systemic antineoplastic regimens, and delineate the role of clinical pharmacists in optimizing treatment strategies and maternal-fetal outcomes.

## 2 Data and methods

**Figure 1:**
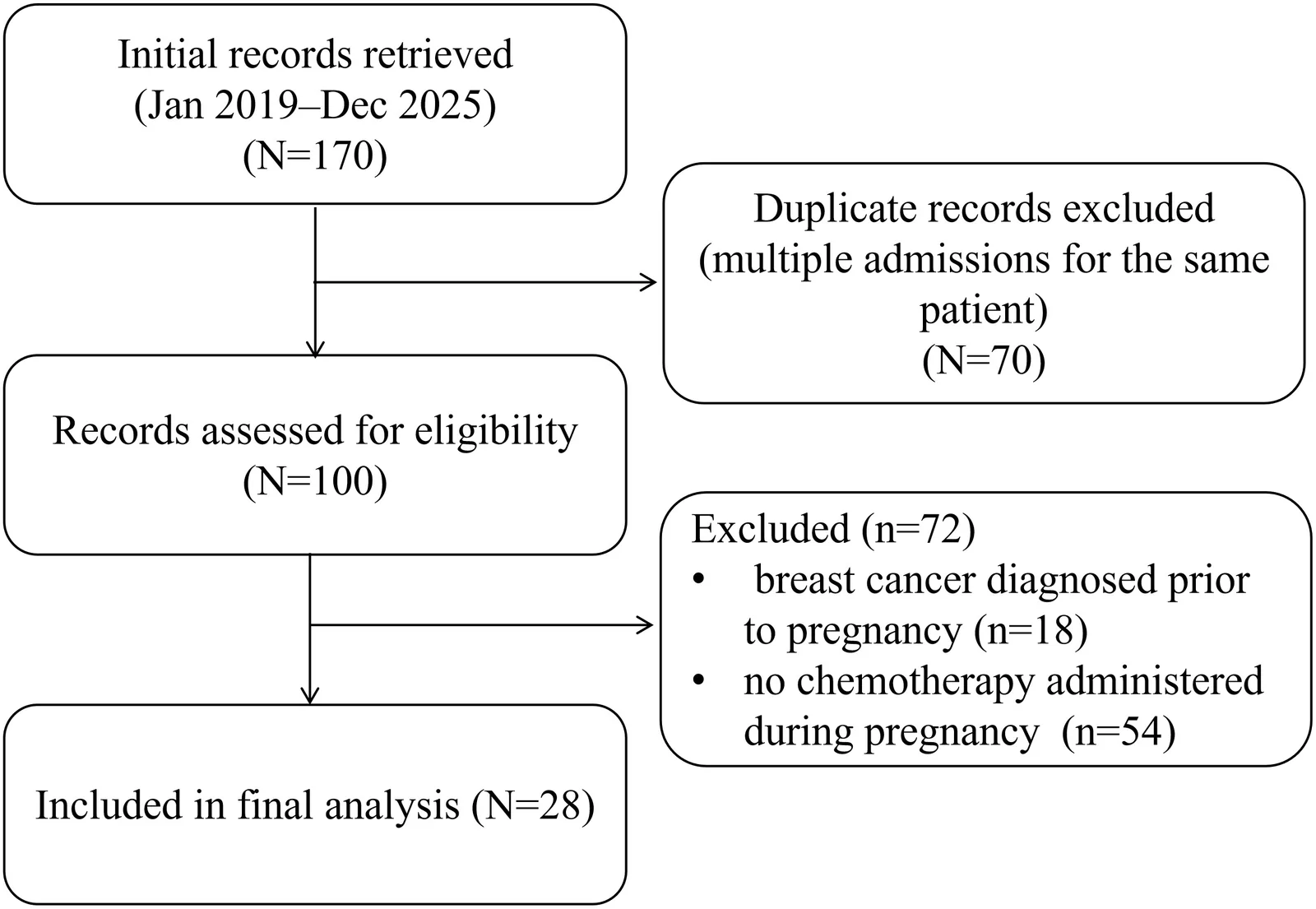
Participant flow diagram of the present cross-sectional study. Deduplication was performed based on medical record number, patients with multiple admissions were consolidated into a single case.

This retrospective, single-center, cross-sectional study included patients diagnosed with PABC between January 2019 and December 2025. This study adopted consecutive sampling, enrolling all eligible patients meeting the inclusion criteria during the study period (**Figure 1**). No *a priori* power calculation was performed for sample size determination, given the descriptive nature of this study and the rarity of pregnancy-associated breast cancer. All enrolled patients represent a complete sample from a single center. Detailed information on chemotherapy regimens administered during pregnancy, pregnancy outcomes, and neonatal adverse events (defined as events occurring within 28 days after birth) was extracted from the hospital electronic database. Inclusion criteria were: (1) age ≥18 years; (2) confirmed breast cancer diagnosis; (3) receipt of chemotherapy during pregnancy; and (4) documented pregnancy completion regardless of outcome. Exclusion criteria were: (1) breast cancer diagnosed prior to pregnancy; and (2) no chemotherapy administered during pregnancy. Data extracted from the Hospital Information System (HIS, system manufacturer: Fugle.Publics.cWinForm) at the Obstetrics and Gynecology Hospital of Fudan University, included: age, weight, family history (genetic disorders and breast cancer), biopsy method, breast cancer stage and molecular subtype, gestational age at diagnosis and at chemotherapy initiation, clinical diagnosis, MDT recommendations, chemotherapy regimen and cycle number, premedication protocol and dosages, occurrence and types of chemotherapy-related adverse reactions, monitoring indicators (complete blood count, liver and renal function, electrocardiogram), gestational age at chemotherapy cessation, mode of delivery, gestational age at delivery, birth weight, Apgar score, and fetal ultrasound findings for growth and development. Data were extracted from the HIS by two independent investigators, with discrepancies resolved through consensus.

The age threshold of 18 years was applied to exclude minor patients, who have distinct ethical and clinical management schemes and may introduce population heterogeneity. Only patients with definite pathological diagnosis of breast cancer and complete gestational chemotherapy records were included to guarantee reliable exposure data, and cases with complete pregnancy follow-up data were selected to fully collect neonatal safety endpoints.

This represents a convenience sample of all eligible patients treated during the study period; no *a priori* power calculation was performed given the retrospective descriptive nature of the study. This research center is a tertiary referral hospital, and the characteristics of referral may limit the promotion of the results of this study.

Two researchers extracted data independently and conducted cross-check validation to resolve data discrepancies. Complete case analysis was applied to variables with low missing rates, while multiple imputation was used for key outcome indicators.

All patients were managed through an MDT care pathway involving specialists from breast surgery, obstetrics, neonatology, pharmacy, pathology, radiology, ultrasound, and other relevant disciplines, including clinical pharmacists. Weekly MDT meetings were held on Friday mornings, with *ad hoc* emergency consultations available during ward rounds when necessary, to review treatment plans, optimize medication regimens, manage adverse events, and coordinate obstetric care.

Statistical analysis was performed using IBM SPSS Statistics (version 25.0). Normality tests were conducted on all continuous variables; Normal distribution data is represented by mean ± standard deviation, and independent sample t-test is used for inter group comparison. Non normally distributed data are represented by median (quartiles) and analyzed using Mann Whitney U test. The categorical data is summarized by count and percentage, and inter group comparison is performed using Fisher’s exact probability method based on sample size and theoretical frequency. Normality of continuous variables was assessed using the Shapiro-Wilk test; continuous data are presented as mean ± SD for normally distributed data and median (IQR) for non-normally distributed data. This cross-sectional study assessed the normality of all continuous variables using the Shapiro-Wilk test combined with Q-Q plots. Normally distributed continuous variables were compared between subgroups via independent-samples t-test (parametric test), while non-normally distributed continuous variables were analyzed using the Mann-Whitney U test (non-parametric test).

Given the descriptive design and small sample size of this retrospective analysis, formal multivariable adjustment for confounders was not conducted in the current study. Major potential confounders including maternal age, gestational age at tumor diagnosis, and tumor stage should be incorporated into multivariate regression models in future larger-scale investigations to minimize confounding bias.

This study was approved by the Ethics Committee of the Obstetrics and Gynecology Hospital of Fudan University (Approval No. 2026-64). The study was conducted in accordance with the Declaration of Helsinki and applicable local legislation and institutional requirements. Due to its retrospective nature, the Ethics Committee granted a waiver of informed consent.

A major limitation of the present study is the small sample size of only 28 patients. The limited cohort substantially reduces statistical power for subgroup stratification and multivariate adjustment, and restricts the generalizability of our findings to all patients with the corresponding disease. Future multicenter investigations with larger sample populations are warranted to confirm our observations. We only report descriptive statistics in this study. No inferential statistical testing was performed for two key reasons: first, this work is a purely descriptive observational study without pre-specified comparative research hypotheses; second, the limited sample size may compromise the validity and stability of inferential analyses. All results presented herein are only used to characterize the baseline features of the included population, and readers should avoid drawing comparative or causal inferences from these descriptive data.

## 3 Results

### 3.1 Demographic characteristic

A total of 28 pregnant patients with breast cancer, all diagnosed by core needle biopsy, received chemotherapy during pregnancy. The mean age at diagnosis was 32.41 ± 3.80 years. Diagnosis was made during the second trimester in 64.29% of patients, and stage II was the most common tumor stage, accounting for 39.29% (**Table 1**). Among the 28 patients, ER/PR-positive cases accounted for 46.43% (13/28), and HER2-positive for 78.57% (22/28).

**Table 1:** Demographic and clinical characteristics.

Patient characteristics	Proportion (n/N)
Age at diagnosis (years)	
26-30	21.43% (6/28)
31-35	64.29% (18/28)
36-40	7.14% (2/28)
41-45	7.14% (2/28)
Mean age	32.41 ± 3.80
Family history of hereditary disease	
Diabetes	7.14% (2/28)
Gastric cancer	7.14% (2/28)
Cervical cancer	7.14% (2/28)
Esophageal cancer	3.57% (1/28)
Family history of breast cancer	
None	0% (0)
Biopsy method	
Core needle biopsy	100% (28/28)
Gestational age at breast cancer diagnosis	
Second trimester, 14–27 6/7 weeks	64.29% (18/28)
Third trimester, ≥28 weeks	35.71% (10/28)
Clinical stage	
Stage I	3.57% (1/28)
Stage II	39.29% (11/28)
Stage III	21.43% (6/28)
Unknown	35.71% (10/28)
Hormonal status	
Positive	46.43% (13/28)
Negative	39.29% (11/28)
Unknown	14.29% (4/28)
Her2 status	
Positive	78.57% (22/28)
Negative	7.14% (2/28)
Unknown	14.29% (4/28)

### 3.2 Choice of chemotherapy regimen

Chemotherapy was initiated from the second trimester onward in 64.29% of patients. The most common regimen was EC-T (epirubicin plus cyclophosphamide followed by sequential taxane), accounting for 39.29% of cases. The dosage of anti-tumor drugs is calculated based on the patient’s body surface area. Among these, one patient developed grade III myelosuppression after the second paclitaxel dose, leading to suspension following MDT review. Two patients were intolerant to paclitaxel and were switched to nanoparticle albumin-bound paclitaxel after MDT discussion. All patients received appropriate premedication tailored to the specific agents, including oral dexamethasone (46.43%) and intravenous ondansetron (50.00%). Chemotherapy-related adverse events were comparable to those in non-pregnant patients, including leukopenia (7.14%), severe myelosuppression (7.14%), nausea (3.57%), facial flushing (3.57%), and thrombocytopenia (3.57%) (**Table 2**).

**Table 2:** Chemotherapy regimens and gestational age in PABC.

Treatment regimen information	Proportion (n/N)
Gestational age at chemotherapy initiation	
Second trimester, 14–27 6/7 weeks	64.29% (18/28)
Third trimester, ≥28 weeks	21.43% (6/28)
Postpartum chemotherapy	14.29% (4/28)
Chemotherapy regimen	
EC-T	39.29% (11/28)
EC+wP	25% (7/28)
EC	14.29% (4/28)
T-EC	7.14% (2/28)
TC	3.57% (1/28)
P	3.57% (1/28)
nab-P-Cb	3.57% (1/28)
PLD-nab-P	3.57% (1/28)
Taxanes used during pregnancy	
Docetaxel	50.00% (14/28)
Paclitaxel	21.43% (6/28)
Nanoparticle albumin-bound paclitaxel	7.14% (2/28)
Chemotherapy premedication (calculated per patient; if a patient received a drug multiple times, counted as 1)	
Ondansetron injection	50.00% (14/28)
Dexamethasone tablets	46.43% (13/28)
Diphenhydramine tablets	17.86% (5/28)
Dexamethasone injection	7.14% (2/28)
Ondansetron tablets	7.14% (2/28)
Chemotherapy-related adverse reactions	
Leukopenia	7.14% (2/28)
Severe myelosuppression	7.14% (2/28)
Nausea	3.57% (1/28)
Facial flushing	3.57% (1/28)
Thrombocytopenia	3.57% (1/28)

E: epirubicin; C: cyclophosphamide; T: docetaxel; wP: weekly paclitaxel; nab-P-Cb: nanoparticle albumin-bound paclitaxel plus carboplatin; PLD-nab-P: pegylated liposomal doxorubicin plus nanoparticle albumin-bound paclitaxel; DXM: dexamethasone; Leukopenia: white blood cell<4.0 *10^9^/L; Severe myelosuppression: white blood cell< 1.0*10^9^/L, neutrophil < 0.5*10^9^/L, platelet< 25*10^9^/L, hemoglobin< 65g/L, patients meeting any of the above criteria are defined as having Severe myelosuppression; Thrombocytopenia: platelet< 100*10^9^/L.

Adverse reactions were assessed and classified according to the Common Terminology Criteria for Adverse Events (CTCAE). The recorded adverse event categories were defined as follows:

1. Hematologic toxicity: neutropenia, anemia, thrombocytopenia and myelosuppression confirmed by routine blood tests; 2. Gastrointestinal toxicity: nausea, vomiting, diarrhea, oral mucositis and anorexia; 3. Hepatic and renal impairment: abnormal liver or renal function indicators identified via biochemical examination; 4. Neurotoxicity: peripheral numbness, paresthesia and other neurological discomforts related to chemotherapy; 5. Cutaneous adverse events: rash, pigmentation and skin erythema; 6. Electrolyte disturbances: hypokalemia, hypomagnesemia and other metabolic abnormalities.

### 3.3 Pregnancy outcomes

Cesarean section was performed in 78.57% of patients, and 50.00% delivered at term. The mean neonatal birth weight was 2,730 ± 497 g, with 28.57% of neonates classified as low birth weight. Five neonates (17.86%) required Neonatal Intensive Care Unit (NICU) admission. Adverse neonatal outcomes included postnatal tachypnea with retractions in two (7.14%), flaccid limbs with ruddy complexion and acrocyanosis in one, bilateral preauricular tags in one (5 mm and 1 mm on the left, 2 mm on the right), and recurrent vomiting in one (**Table 3**).

**Table 3:** Pregnancy outcomes.

Pregnancy outcome	Proportion (n/N)
Delivery mode	
Cesarean section	78.57% (22/28)
Vaginal delivery	14.29% (4/28)
Unknown	7.14% (2/28)
Gestational age at delivery	
Early preterm, <32 weeks	3.57% (1/28)
Moderate to late preterm, 32–36 weeks	32.14% (9/28)
Full term, ≥37 weeks	50.00% (14/28)
Unknown	14.29% (4/28)
Mean birth weight (g)	2730 ± 497
Low birth weight, <2,500 g	28.57% (8/28)
NICU admission	17.86% (5/28)
Apgar score <7 at 1 minute	3.57% (1/28)
Apgar score <7 at 5 minutes	0% (0/28)
Neonatal adverse events	
Postnatal tachypnea with retractions	7.14% (2/28)
Flaccid limbs, ruddy complexion with acrocyanosis	3.57% (1/28)
Bilateral preauricular tags (left: 5 mm, 1 mm; right: 2 mm)	3.57% (1/28)
Recurrent vomiting	3.57% (1/28)

Low birth weight<2,500 g; NICU admission: 1. Preterm infants and low birth weight infants: very low birth weight infants (<1500 g), extremely low birth weight infants (<1000 g), and extremely preterm infants requiring close monitoring; Hypoxic-ischemic disorders: severe hypoxic-ischemic encephalopathy (HIE), hypoxia-induced brain injury, neonatal asphyxia (requiring post-resuscitation monitoring), etc.; Respiratory diseases: infants with acute respiratory failure and lung injury of various etiologies who require respiratory support such as endotracheal intubation, mechanical ventilation and CPAP; Neurological diseases: intracranial hemorrhage, severe intracranial lesions, etc.; Infection and shock: severe infection, septic shock, sepsis, etc.; Cardiovascular and hematological diseases: refractory arrhythmia, persistent pulmonary hypertension of the newborn, severe water-electrolyte and acid-base disturbances, severe hyperbilirubinemia (e.g., bilirubin encephalopathy), etc.; Other critical illnesses: complex congenital heart disease, inherited metabolic diseases, various birth defects and congenital malformations requiring surgical treatment, and infants with multiple organ dysfunction involving the heart, kidneys, disseminated intravascular coagulation (DIC), etc.

## 4 Discussion

### 4.1 Chemotherapy strategies and drug selection for pregnancy-associated breast cancer under the MDT model

The Multidisciplinary Team (MDT) model has emerged as the cornerstone for the standardized management of Pregnancy-Associated Breast Cancer (PABC). For the cases enrolled in this study, the MDT conducted comprehensive evaluations integrating the gestational age, molecular subtype of the tumor, and fetal safety. Clinical pharmacists played an integral role in furnishing the latest evidence-based pharmacological data—such as placental transfer rates and fetal safety profiles—to optimize the timing and dosage of administration to ensure treatment efficacy while protecting maternal and neonatal health.

A special report by the U.S. National Toxicology Program (NTP) demonstrated that the incidence of fetal malformations following chemotherapy exposure during the first trimester was 14%, whereas exposure during the second and third trimesters decreased to 3.9%, a rate no statistically different from the 3% malformation rate of the general population (11, 12). These data suggest that the teratogenic risk of chemotherapy is significantly reduced when administered during the second and third trimesters, providing a safety rationale for initiating chemotherapy after the first trimester in this study. The 2025 RCOG Guidelines state that chemotherapy is contraindicated during the first trimester but may be used under strict monitoring during the second and third trimesters (13). Accordingly, all prenatal chemotherapy courses in this study were initiated after the first trimester in accordance with this recommendation. To reduce the risk of maternal-fetal myelosuppression and infection, studies recommend that prenatal chemotherapy should be discontinued 2–3 weeks prior to the planned delivery (typically between gestational weeks 35–37) to allow time for the recovery of maternal and fetal bone marrow function (13, 14). In this study, all prenatal chemotherapy courses were completed before 35–37 weeks of gestation, consistent with this safety strategy. Current evidence suggests that outcomes for PABC patients receiving chemotherapy during pregnancy are comparable to, or even better than, those of non-pregnant patients with similar age, stage, and subtype (15). However, the placental transfer of cytotoxic agents remains a critical factor. While nearly all cytotoxic agents cross the placenta, transfer rates vary significantly (16): doxorubicin 4%-7.5% (17, 18), epirubicin 3.7% (19), paclitaxel 1.5% (18, 20), docetaxel 4% (18), cyclophosphamide (active metabolite) 25% (17, 18), and carboplatin 4%–13% (18). Although the placenta can metabolize and excrete these drugs, the process requires at least 3 weeks. Consequently, guidelines recommend monitoring fetal weight and amniotic fluid volume every 3 weeks to identify complications such as Fetal Growth Restriction (FGR) (21).

Regarding drug selection strategies, the combination of anthracyclines and cyclophosphamide represents the most widely utilized regimen during pregnancy, supported by robust evidence-based data. Large-scale international cohort studies have provided critical benchmarks for comparing maternal and neonatal outcomes across various chemotherapy protocols. One 20-year study, which enrolled 1,170 patients with gestational malignancies (including breast, cervical, ovarian, and gastrointestinal cancers, as well as lymphoma and leukemia), analyzed 429 patients who received chemotherapy during pregnancy. In this cohort, utilization rates were 78% for anthracyclines, 69% for alkylating agents (excluding platinum), and 20% for taxanes (22). This research revealed that, compared to anthracyclines, taxanes were associated with increased odds ratios for adverse outcomes such as preterm premature rupture of membranes (PPROM), preterm labor, small-for-gestational-age (SGA) infants, and increased NICU admission rates (22). This suggests that taxanes may elevate the risk for specific complications. Another British study focusing on PABC enrolled 37 patients treated with chemotherapy; the majority (31 cases) received anthracycline-based systemic therapy, with 26 receiving cyclophosphamide and 15 receiving taxanes. At the time, the FEC regimen (Fluorouracil + Epirubicin + Cyclophosphamide) was the most frequent combination (10 cases) (23).

The chemotherapy selection in our study aligns with both the aforementioned research and the 2026 NCCN Guideline recommendations. Anthracycline plus cyclophosphamide-based regimens accounted for 85.72% of our cases. Sequential taxane administration reached 64.29%, reflecting a rapid clinical shift in response to updated guidelines. Since modern protocols have largely excluded fluorouracil from the standard of care, taxanes have become more prevalent in contemporary practice. The discrepancy between our study and the earlier British study is primarily attributable to their earlier data collection period during which fluorouracil-based regimens were still the standard. Our real-world data serves as a practical supplement to current guideline implementation. Furthermore, we observed no serious maternal or neonatal adverse events directly attributable to chemotherapy, reinforcing the relative safety profile of chemotherapy during pregnancy.

### 4.2 Premedication protocols for chemotherapy in pregnancy-associated breast cancer

Guidelines indicate that the combination of anthracyclines and cyclophosphamide carries a high emetogenic risk (24). With regard to antiemetic therapy during pregnancy, 5-HT3 receptor antagonists (e.g., ondansetron) are classified as second-line agents (25), and dexamethasone (DXM) is considered a third-line option (26). The fetal safety of ondansetron remains a subject of epidemiological controversy. Whereas early studies suggested that first-trimester exposure might increase the risk of fetal cardiac defects and oral clefts (27, 28), recent sensitivity analyses—after adjusting for confounding factors—have found no significant association with adverse pregnancy outcomes (29). As a result, clinical use requires a careful risk-benefit assessment and continuous monitoring of updated evidence. Despite these concerns, ondansetron is commonly used during pregnancy for chemotherapy-induced nausea and vomiting, and is considered an acceptable antiemetic alternative in this setting (6).

The placental transfer characteristics of DXM are a critical consideration in formulating PABC chemotherapy protocols. Due to the limited metabolic capacity of placental 11β-hydroxysteroid dehydrogenase type 2 (11β-HSD2) toward DXM, the drug is not fully inactivated within the placenta, allowing approximately 14.4% ± 1.5% of the maternal dose to cross the placental barrier into the fetal circulation (30–32), thereby increasing the risk of potential fetal exposure. This pharmacological profile poses a unique challenge for clinical premedication: standard protocols for docetaxel require oral DXM starting one day prior to infusion (8 mg twice daily for three days). However, repeated exposure to high-dose corticosteroids may exacerbate maternal adverse reactions and pose potential risks to the fetus. Based on these concerns, the clinical pharmacist raised a core question during the MDT process: Could the DXM administration strategy be optimized to safeguard efficacy while minimizing maternal-fetal risk? Evidence-based literature provides strong support for such an optimization strategy. A retrospective analysis of 82 patients with early-stage breast cancer showed that either a single intravenous dose of 20 mg DXM or a 12 mg intravenous dose combined with three days of 4 mg oral DXM post-chemotherapy improved patient compliance and reduced treatment delays without increasing docetaxel-related toxicity (33). A Phase I dose-escalation study further confirmed that reducing DXM to a 3-day oral regimen (4mg – 8mg – 4mg) for docetaxel premedication did not increase the risk of grade III/IV hypersensitivity reactions or fluid retention (34). Additionally, a randomized controlled trial demonstrated that oral DXM at 4.5 mg once daily was equivalent in efficacy and safety to 8 mg twice daily for preventing hypersensitivity (35). In summary, optimized, reduced-dose DXM premedication has been proven safe and effective in non-pregnant patients. Integrating the placental transfer properties of DXM with the principle of minimal drug exposure during pregnancy, this study proposed a modified regimen: DXM 8 mg once daily for 3 days. Compared to the standard protocol (total dose of 48 mg), this approach reduces total DXM exposure by 50%. Theoretically, this regimen maintains therapeutic efficacy while lowering fetal glucocorticoid exposure; however, its specific risk-benefit balance in PABC patients warrants further validation through prospective studies.

### 4.3 Effects of PABC chemotherapy exposure on perinatal outcomes and long-term development

This study evaluated the perinatal outcomes of neonates born to PABC patients who received chemotherapy during pregnancy. The results are broadly consistent with existing literature and further contribute to the clinical evidence on short-term adverse events and long-term developmental outcomes in this population.

In terms of obstetric outcomes, research indicates that the malformation rate among children born to PABC patients does not significantly differ from that of unexposed children (4.0% vs. 4.1%). However, the risk of cesarean delivery is significantly elevated, particularly regarding planned cesarean deliveries (35.4% vs. 8.3%), a difference primarily driven by clinical decisions to initiate oncological treatment as early as possible (36). In this study, cesarean deliveries accounted for 78.57% of deliveries; notably, all preterm and low-birth-weight (LBW) infants were delivered via cesarean section. This finding aligns with the reported trend of increased cesarean risk, suggesting that active iatrogenic intervention (such as planned cesarean deliveries) may be a necessary strategy for PABC patients.

With regard to neonatal adverse events, this study observed multisystem involvement, including the following: respiratory complications — tachypnea with inspiratory retractions (7.14%); neurological manifestations — limb hypotonia accompanied by cyanosis and Apgar scores of < 7 (3.57%); and digestive symptoms — repeated vomiting (3.57%). The NICU admission rate reached 17.86%. Consistent with the report by Loibl et al. (37), these findings suggest that neonates of PABC patients may face a risk of multisystem involvement. It is noteworthy that one case of bilateral preauricular tags was identified in this study (two lesions measuring 5 mm and 1 mm on the left side, and one lesion measuring 2 mm on the right side). Preauricular tags are common minor congenital malformations resulting from first branchial arch developmental anomalies occurring during the 3rd to 8th week after fertilization (the critical period of organogenesis). Existing literature suggests that their development is primarily associated with the hormonal milieu of pregnancy and individual susceptibility, with no established causal link to chemotherapeutic agents (38). We conducted a systematic search of PubMed, Embase, and the OpenVigil FDA database and found no published reports associating epirubicin, paclitaxel, or cyclophosphamide with skin tags or preauricular tags. Although one case of cyclophosphamide-associated acrochordon was recorded in the OpenVigil FDA database (39), disproportionality analysis showed no significant correlation. Therefore, the preauricular tags in this case are more likely an incidental congenital variant rather than a chemotherapy-related finding. Nevertheless, our findings still suggest that neonates born to PABC patients should undergo careful physical examination after delivery to enable early detection of potential minor malformations.

With respect to the impact of chemotherapy on fetal development, existing studies show that chemotherapy is associated with increased risks of fetal growth restriction (FGR) (21%), preterm birth (52%, with spontaneous preterm birth accounting for 9%), and low birth weight (LBW). The earlier chemotherapy is initiated, the greater the negative impact on fetal growth (40). Ultrasound monitoring has confirmed that parameters such as estimated fetal weight, head circumference, abdominal circumference, and femur length decline continuously with cumulative chemotherapy exposure. Furthermore, for a fixed treatment course, a later gestational age at initiation of chemotherapy has a more pronounced negative effect on fetal growth indicators, suggesting differential sensitivity: the embryo is highly sensitive during the first trimester, whereas chemotherapy in the second and third trimesters may inhibit growth by affecting placental function (41). While anthracyclines show no strong direct correlation with FGR or small-for-gestational-age (SGA) status, taxanes and platinum agents increase the proportion of SGA infants (42). In this study, preterm infants accounted for 35.71% of cases and LBW infants for 28.57%, consistent with the high incidence of impaired growth reported in the literature, suggesting that the cumulative effect of chemotherapy significantly interferes with normal intrauterine development.

With regard to long-term prognosis, current limited evidence suggests that children of PABC patients with prenatal chemotherapy appear to show no significant differences in neurological or cardiac development or function compared with unexposed children (43). Furthermore, chemotherapy during pregnancy is not associated with an increased risk of dental abnormalities (44). These findings provide a vital reference for clinical decision-making: when weighing the short-term fetal risks against the benefits of maternal treatment, the reassuring long-term safety profile must also be integrated into the evaluation.

On the basis of these considerations, clinical pharmacists within the MDT framework delivered targeted health education focused on neonatal safety. Patients were clearly informed that short-term risks (e.g., preterm birth, LBW) are manageable and that maternal-fetal safety can be secured through multidisciplinary collaboration. It was explained that chemotherapy administered in the second and third trimesters does not significantly increase the risk of fetal malformations. It was noted that taxanes and platinum agents may inhibit fetal growth, necessitating ongoing monitoring via prenatal ultrasound and postnatal follow-up. Finally, it was emphasized that long-term neurodevelopmental and cardiac outcomes are comparable to those of the general pediatric population, thereby effectively alleviating patient anxiety.

In summary, chemotherapy for pregnancy-associated breast cancer (PABC) is associated with increased risks of preterm birth, cesarean section, and LBW, with short-term neonatal adverse events presenting across multiple organ systems. Although certain chemotherapeutic agents may inhibit fetal growth, the available literature indicates no significant long-term adverse effects on neurological and cardiac function. Within the framework of the MDT model, clinical pharmacists contribute to regimen formulation and comprehensive management, ensuring maternal anti-tumor efficacy while collaborating with obstetric and neonatal specialists to minimize exposure risks and optimize outcomes for both mother and child.

## 5 Conclusion

This study conducted a retrospective analysis of chemotherapy safety and maternal-fetal outcomes in patients with pregnancy-associated breast cancer under the multidisciplinary team (MDT) model. The results suggest that, in this small sample, anthracycline-based chemotherapy administered during the second and third trimesters appeared to be associated with acceptable safety profiles and maternal-fetal outcomes. In this process, clinical pharmacists were involved in regimen optimization, adverse reaction management, and medication education, reflecting the operational framework of MDT collaboration in the management of pregnancy-associated breast cancer. However, the retrospective single-center design, small sample size, absence of a comparator group, and lack of long-term pediatric follow-up limit the generalizability and causal inference of these findings. Future multicenter prospective studies with standardized pharmacist intervention protocols and extended offspring follow-up are warranted.

## 6 Limitations

This study has several limitations. First, the retrospective, single-center design, small sample size (n=28), and lack of randomization limit the statistical power and generalizability of our findings, and preclude causal inference. Second, the absence of a comparator group of PABC patients managed without pharmacist participation prevents definitive assessment of the incremental benefit attributable to pharmacist interventions. Third, data quality was constrained by missing information: clinical stage at diagnosis was unavailable for 35% of patients due to incomplete transfer of external records and, in some cases, patient refusal of staging workup during pregnancy over fetal radiation concerns; long-term maternal oncologic outcomes were incomplete because a considerable proportion of patients received follow-up at other institutions; and pediatric developmental data beyond the neonatal period were not collected. Fourth, we did not conduct a formal assessment of pharmacist intervention fidelity, which limits our ability to evaluate the consistency of pharmaceutical care delivery. Additionally, no adjustment for potential confounders (e.g., maternal age, gestational age at diagnosis, tumor stage) was performed, which may have introduced bias in the interpretation of the observed associations. Finally, the findings may have limited generalizability to settings with different healthcare infrastructures, pharmacist staffing models, or patient populations. Future multicenter prospective studies with comparator groups, standardized intervention protocols, and extended pediatric follow-up are warranted to validate these preliminary findings.

## Data Availability

The original contributions presented in the study are included in the article. Further inquiries can be directed to the corresponding authors.

## References

[1] Akhlaqi M Ghofrani A Najdi N Ranjkesh M Almasi-Hashiani A . A systematic review and meta-analysis of pregnancy-associated breast cancer incidence rate. BMC Cancer. (2025) 25:660. doi: 10.1186/s12885-025-14091-2 40211262 PMC11987432

[2] Basaran D Turgal M Beksac K Ozyuncu O Aran O Beksac MS . Pregnancy-associated breast cancer: clinicopathological characteristics of 20 cases with a focus on identifiable causes of diagnostic delay. Breast Care (Basel). (2014) 9:355–9. doi: 10.1159/000366436 25759617 PMC4322684

[3] Schedin P . Pregnancy-associated breast cancer and metastasis. Nat Rev Cancer. (2006) 6:281–91. doi: 10.1038/nrc1839 16557280

[4] Suelmann BBM van Dooijeweert C Bakhuis CFJ Linn S van der Wall E van Diest PJ . Pregnancy-associated breast cancer: the influence of gestational age. Endocr Relat Cancer. (2022) 29:129–38. doi: 10.1530/ERC-21-0206 34935632

[5] Zhang R Liu X Huang W Shao B Yan Y Liang X . Clinicopathological features and prognosis of patients with pregnancy-associated breast cancer: a matched case control study. Asia Pac J Clin Oncol. (2021) 17:396–402. doi: 10.1111/ajco.13528 33655647 PMC8359456

[6] Duggirala N Zhang S Master A Rao R Kapoor NS Bardia A . Biology, care, and outcomes of gestational breast cancers: a review. Breast Cancer Res Treat. (2025) 211:547–59. doi: 10.1007/s10549-025-07684-9 40155574 PMC12031940

[7] Huis In 't Veld EA Van Assche IA Amant F . Long-term outcomes of children after prenatal exposure to maternal cancer and its treatment. Acta Obstet Gynecol Scand. (2024) 103:757–60. doi: 10.1111/aogs.14811 38419133 PMC10993341

[8] National Comprehensive Cancer Network . NCCN Clinical Practice Guidelines in Oncology: Breast Cancer. Version 2.2026 (2026). Available online at: https://www.nccn.org/guidelines/guidelines-detail?category=1&id=1419 (Accessed April 27, 2026).

[9] Pariente G Leibson T Carls A Adams-Webber T Ito S Koren G . Pregnancy-associated changes in pharmacokinetics: A systematic review. PloS Med. (2016) 13:e1002160. doi: 10.1371/journal.pmed.1002160 27802281 PMC5089741

[10] Gao Z Song C Li G Lin H Lian X Zhang N . Pyrotinib treatment on HER2-positive gastric cancer cells promotes the released exosomes to enhance endothelial cell progression, which can be counteracted by apatinib. Onco Targets Ther. (2019) 12:2777–87. doi: 10.2147/OTT.S194768 31114227 PMC6489591

[11] National Toxicology Program . NTP monograph: developmental effects and pregnancy outcomes associated with cancer chemotherapy use during pregnancy. NTP Monogr. (2013) (2):i–214. 24736875

[12] Honein MA Kirby RS Meyer RE Xing J Skerrette NI Yuskiv N . The association between major birth defects and preterm birth. Matern Child Health J. (2009) 13:164–75. doi: 10.1007/s10995-008-0348-y 18484173 PMC11669608

[13] Armstrong A Gandhi A Frank S Williams D Nimalasena S Royal College of Obstetricians and Gynaecologists . Pregnancy and breast cancer: green-top guideline no. 12. BJOG. (2025) 132:e194–228. doi: 10.1111/1471-0528.18270 40855616

[14] Bajpai J Simha V Shylasree TS Sarin R Pathak R Popat P . Pregnancy associated breast cancer (PABC): Report from a gestational cancer registry from a tertiary cancer care centre, India. Breast. (2021) 56:88–95. doi: 10.1016/j.breast.2021.02.005 33640524 PMC7933532

[15] Johnson HM Song J Warneke CL Martinez AL Litton JK Oke OC . Outcomes of patients treated with chemotherapy for breast cancer during pregnancy compared with nonpregnant breast cancer patients treated with systemic therapy. Cancer. (2025) 131:e35619. doi: 10.1002/cncr.35619 39470464 PMC11784491

[16] Cardonick E Iacobucci A . Use of chemotherapy during human pregnancy. Lancet Oncol. (2004) 5:283–91. doi: 10.1016/S1470-2045(04)01466-4 15120665

[17] Van Calsteren K Verbesselt R Beijnen J Devlieger R De Catte L Chai DC . Transplacental transfer of anthracyclines, vinblastine, and 4-hydroxy-cyclophosphamide in a baboon model. Gynecol Oncol. (2010) 119:594–600. doi: 10.1016/j.ygyno.2010.08.019 20846713

[18] Benoit L Mir O Vialard F Berveiller P . Cancer during pregnancy: A review of preclinical and clinical transplacental transfer of anticancer agents. Cancers (Basel). (2021) 13:1238. doi: 10.3390/cancers13061238 33799824 PMC8000411

[19] Gaillard B Leng JJ Grellet J Ducint D Saux MC . Passage transplacentaire de l'épirubicine [Transplacental passage of epirubicin. J Gynecol Obstet Biol Reprod (Paris). (1995) 24:63–8. 7730570

[20] Calsteren KV Verbesselt R Devlieger R De Catte L Chai DC Van Bree R . Transplacental transfer of paclitaxel, docetaxel, carboplatin, and trastuzumab in a baboon model. Int J Gynecol Cancer. (2010) 20:1456–64. doi: 10.1111/IGC.0b013e3181fb18c8 21307819

[21] Tan A Wang W Long C Zhang Z Ngeow J Mattar C . Pregnancy-associated breast cancer: Management of the mother, fetus and tumour. Ann Acad Med Singap. (2025) 54:235–46. doi: 10.47102/annals-acadmedsg.2024320 40324891

[22] de Haan J Verheecke M Van Calsteren K Van Calster B Shmakov RG Mhallem Gziri M . Oncological management and obstetric and neonatal outcomes for women diagnosed with cancer during pregnancy: a 20-year international cohort study of 1170 patients. Lancet Oncol. (2019) 19:337–46. doi: 10.1016/S1470-2045(18)30059-7 29395867

[23] Hardy C Brand A Jones J Knight M Banfield P . The UK Breast Cancer in Pregnancy (UKBCiP) Study. Incidence, diagnosis, management and short-term outcomes of breast cancer first diagnosed during pregnancy in the United Kingdom: A population-based descriptive study. NIHR Open Res. (202) 4:40. doi: 10.3310/nihropenres.13652.2 39233778 PMC11372348

[24] Herrstedt J Clark-Snow R Ruhlmann CH Molassiotis A Olver I Rapoport BL . 2023 MASCC and ESMO guideline update for the prevention of chemotherapy- and radiotherapy-induced nausea and vomiting. ESMO Open. (2024) 9:102195. doi: 10.1016/j.esmoop.2023.102195 38458657 PMC10937211

[25] Nelson-Piercy C Dean C Shehmar M Gadsby R O'Hara M Hodson K . The management of nausea and vomiting in pregnancy and hyperemesis gravidarum (Green-top guideline no. 69). BJOG. (2024) 13:e1–e30. doi: 10.1111/1471-0528.17739 38311315

[26] Gerede A Stavros S Moustakli E Potiris A Orgianelis I Zikopoulos A . Hyperemesis in pregnancy: complications and treatment. Med Sci (Basel). (2025) 13:132. doi: 10.3390/medsci13030132 40843754 PMC12372004

[27] Danielsson B Wikner BN Källén B . Use of ondansetron during pregnancy and congenital malformations in the infant. Reprod Toxicol. (2014) 50:134–7. doi: 10.1016/j.reprotox.2014.10.017 25450422

[28] Anderka M Mitchell AA Louik C Werler MM Hernández-Diaz S Rasmussen SA . Medications used to treat nausea and vomiting of pregnancy and the risk of selected birth defects. Birth Defects Res A Clin Mol Teratol. (2012) 94:22–30. doi: 10.1002/bdra.22865 22102545 PMC3299087

[29] Cao X Sun M Yang Q Wang Q Hou L Wang J . Risk of abnormal pregnancy outcomes after using ondansetron during pregnancy: A systematic review and meta-analysis. Front Pharmacol. (2022) 13:951072. doi: 10.3389/fphar.2022.951072 36120333 PMC9480102

[30] Bondok RS El Sharnouby NM Eid HE Abd Elmaksoud AM . Pulsed steroid therapy is an effective treatment for intractable hyperemesis gravidarum. Crit Care Med. (2006) 34:2781–3. doi: 10.1097/01.CCM.0000242156.15757.70 16957638

[31] Van Der Heijden JEM Van Hove H Van Elst NM Van Den Broek P Van Drongelen J Scheepers HCJ . Optimization of the betamethasone and dexamethasone dosing regimen during pregnancy: a combined placenta perfusion and pregnancy physiologically based pharmacokinetic modeling approach. Am J Obstet Gynecol. (2025) 232:228.e1–9. doi: 10.1016/j.ajog.2024.05.012 38763343

[32] Sloboda DM Challis JR Moss TJ Newnham JP . Synthetic glucocorticoids: antenatal administration and long-term implications. Curr Pharm Des. (2005) 11:1459–72. doi: 10.2174/1381612053507873 15853676

[33] Hager A Kondle S Agarwal A Chintapenta M Horadam R Sadeghi N . Comparative study of dexamethasone premedication regimens with docetaxel chemotherapy in early HER-2 positive breast cancer: A safety net hospital experience. J Oncol Pharm Pract. (2025) 31:236–44. doi: 10.1177/10781552241232692 38425269 PMC11898369

[34] Lugtenberg RT de Groot S Houtsma D Dezentjé VO Vulink AJE Fischer MJ . Phase 1 study to evaluate the safety of reducing the prophylactic dose of dexamethasone around docetaxel infusion in patients with prostate and breast cancer. Cancers (Basel). (2023) 15:1691. doi: 10.3390/cancers15061691 36980577 PMC10046524

[35] Zhang M Chen Z Yang Y Cheng H Li C He Q . Pretreatment with different doses of dexamethasone in the prevention of docetaxel-induced hypersensitivity. Pak J Pharm Sci. (2017) 30:61–5. 28603114

[36] Lundberg FE Gkekos L Rodriguez-Wallberg KA Fredriksson I Johansson ALV . Risk of obstetric and perinatal complications in women presenting with breast cancer during pregnancy and the first year postpartum in Sweden 1973-2017: A population-based matched study. Acta Obstet Gynecol Scand. (2024) 103:684–94. doi: 10.1111/aogs.14555 36959086 PMC10993363

[37] Loibl S Han SN von Minckwitz G Bontenbal M Ring A Giermek J . Treatment of breast cancer during pregnancy: an observational study. Lancet Oncol. (2012) 13:887–96. doi: 10.1016/S1470-2045(12)70261-9 22902483

[38] Bord A Valsky DV Yagel S . Prenatal sonographic diagnosis of congenital perineal skin tag: case report and review of the literature. Prenat Diagn. (2006) 26:1065–7. doi: 10.1002/pd.1557 16952203

[39] OpenVigil FDA . Open Vigil FDA - an Openfda API Based AERS Data Search Engine Pharmacovigilance Tool Made by the Openvigil Project (2011). Available online at: https://ovfda.h2876314.stratoserver.net (Accessed July 20, 2026).

[40] Maggen C Wolters VERA Van Calsteren K Cardonick E Laenen A Heimovaara JH . Impact of chemotherapy during pregnancy on fetal growth. J Matern Fetal Neonatal Med. (2022) 35:10314–23. doi: 10.1080/14767058.2022.2128645 36202393

[41] National Toxicology Program . Actions From Peer Review of the Draft NTP Monograph on Developmental Effects and Pregnancy Outcomes Associated With Cancer Chemotherapy Use During Pregnancy. October 1-2, 2012. Available online at: https://ntp.niehs.nih.gov/sites/default/files/ntp/about_ntp/monopeerrvw/2012/october/actionscancerchemo_508.pdf?f_link_type=f_linkinlinenote&flow_extra=eyJpbmxpbmVfZGlzcGxheV9wb3NpdGlvbiI6MCwiZG9jX3Bvc2l0aW9uIjowLCJkb2NfaWQiOiI1NWNjODkxYTgxMGZiZWY4LWZjYTI2 (Accessed January 5, 2026). 24736875

[42] Aranda-Gutierrez A Ferrigno Guajardo AS Vaca-Cartagena BF Gonzalez-Sanchez DG Ramirez-Cisneros A Becerril-Gaitan A . Obstetric and neonatal outcomes following taxane use during pregnancy: a systematic review. BMC Cancer. (2024) 24:9. doi: 10.1186/s12885-023-11704-6 38166767 PMC10763111

[43] Amant F Van Calsteren K Halaska MJ Gziri MM Hui W Lagae L . Long-term cognitive and cardiac outcomes after prenatal exposure to chemotherapy in children aged 18 months or older: an observational study. Lancet Oncol. (2012) 13:256–64. doi: 10.1016/S1470-2045(11)70363-1 22326925

[44] Iyer NS Trager L Gaughan J Akoto S Cardonick E . Paediatric dental outcomes among children exposed to chemotherapy in utero. Int J Paediatr Dent. (2022) 32:116–22. doi: 10.1111/ipd.12801 33960557

